# Orthopædic Controversy in Paris

**Published:** 1844-03-23

**Authors:** 


### ORTHOPÆDIC CONTROVERSY IN PARIS.

A very angry dispute has, for some time past, been going on between M. *Guerin—the* orthopædist (par excellence) of the French metropolis—and M. *Malgaigne* and some other members of the French Academy, relative to certain statistical reports of alleged cures of various deformities, communicated by the former gentlemen to the Bureau des Hôpitaux. Many of his *confreres* had long suspected that the doctor of the establishment of La Muette, (near Passy, we believe,) in his overflowing admiration of subcutaneous Myotomy and Tenotomy, must surely have somewhat exaggerated the marvellous success which attended all his operations. Wry-necks, crooked backs, bent knees, club-feet, stiffened fingers and toes—not to mention squinting and various other kinds of deformity—seemed to be got rid of in a twinkling, and scarcely ever did we hear of a case that was not cured. Our readers are aware that M. *Guerin* is not only very adroit, but also most persevering and courageous, in carrying out his brilliant 'decouverte' (invention, we should rather call it,) of dividing muscles and tendons, without making more than a puncture through the integuments. On one occasion he divided no fewer than forty muscles or tendons in a single case, and the result was, as a matter of course, that the patient was 'parfaitement gueri.' The success of his operations was so unvarying, that many of the medical men of Paris began to have their doubts as to the complete veracity of the reports; and M. *Malgaigne* in particular seems to have set about a strict enquiry, with the view of ascertaining the exact state of the question. His statements are certainly far from being in accordance with the assertions of M. *Guerin.* Many of the cases reported as cured have been, it is said, scarcely, if at all, benefited by the treatment adopted at La Muette; and, in several instances, the same patient figures more than once on the successful list. We need scarcely allude to another charge that has been brought by some against M. *Guerin.* It is alleged that many of the gratuitous patients, sent by the Bureau des Hôpitaux to the Doctor's establishment, have been made to pay for the plaster-moulds that have been taken of their deformities and the apparatus which have been supplied, and that M. *Guerin* has been no loser on these occasions. As we have no opportunities of knowing the truth of this charge, it is better to keep it entirely out of our view, and to confine ourselves solely to the medical part of the accusation. We were not, we must confess, at all surprised when we first read of the charge of bad faith being brought against the orthopædic doctor.

There has always been such extravagance in his published reports, such unheard of success in everything that he set his hand to, such marvellous cures effected by a *coup-de-main*, and withal such silly affectation of scientific profundity, that we long ago settled in our own minds that all that he said was not gospel. That his reports of successful cases have unquestionably been *inaccurate*, cannot, we suppose, be now denied. He may indeed have imposed upon his own credulity, and it may be that thus he has been unconsciously led to mislead the minds of others by his statements. But then there has been all throughout so loud and magniloquent a boasting, that we almost hesitate to give him the benefit of this charitable interpretation.

While unwilling to exculpate M. *Guerin* from, at least, the charge of most unworthy indiscretion, we must lay part of the blame to the charge of the Royal Academy itself, as some of the practices, connected with the proceedings of this learned body, have always appeared to us to open the door for much accidental, if not wilful misstatements. We allude more particularly to the common occurrence of members reporting cases, while still under treatment. Nay, not only this; but, every now and then, we read of successful operations being pompously announced on the very evening perhaps of the day on which they were performed. Sometimes indeed the case is rendered still more dramatic, by the patient being exhibited to the Academy before and after the surgical treatment—all seemingly for the purpose of giving the operator, and the other members, an opportunity of saying something about themselves.

M. *Guerin*, as might be imagined, is especially fond of such exhibitions. For example, we find, in the account of the proceedings of the Academy on the 18th of July last, that he exhibited a patient, affected with lateral deviation of the spine, on whom he had a few days before performed the operation of tenotomy. "I have," said he, "in this case, divided the *longus dorsi* and part of the *sacrolumbaris* on the right side, and the spinal portion of the *longus dorsi* on the left. As the patient, at the present moment, wants part of the muscles that are necessary for the act of standing, he requires to be supported." "The section of these muscles," the learned doctor continued, "not proving quite sufficient in itself for rectifying the deformity, it is necessary in this case to have recourse to an apparatus, for the purpose of keeping the parts in a normal attitude. Here, therefore, we must act, as we do in cases of club-foot, where we first divide the tendons that are at fault, and afterwards retain the limb motionless and in a proper position for some time. I repeat that this patient is not yet cured, and that I have exhibited him only to prove how innocuous the operation of tenotomy is, and to shew its immediate effects." So much for the *show-man;* now for the remarks of some of his auditors. M. *Bouvier* (a rival orthopædic doctor, we believe) gets up and tells the learned Academy that all, that M. *Guerin* has been saying, is mere fudge; that the patient is not a whit benefited by having had his dorsal muscles cut; and that all the *seeming* improvement in the straitness of the back is altogether owing to the person being shewn off in the reclining position.

As a matter of course, human endurance cannot bear such scandal. Up starts the operator, and denounces, as so many falsehoods, the observations of his learned *confrere.* At the same time, he gives vent to certain pathological axioms on his favourite subject, which we cannot do better than give in his own words. 1. "I regard," says he, "that lateral deviation of the spine is a sub-luxation of the vertebræ, and therefore remediable only in one way, by effecting a certain reduction of the displacement in question;—2, that this reduction is indispensable to neutralise, in part, the influence of the vertical action of the weight of the head and upper part of the body;—3, that, in this respect, deviation of the spine is exactly in the same condition as club-foot, which does not become rectified of itself after the division of the contracted tendons, but which requires to be kept mechanically extended afterwards for some time."

The analogy, which M. *Guerin* here seeks to establish between lateral deviation of the spine and club-foot, is, in our opinion, quite faulty and fallacious. There is no permanent contraction of any of the muscles in the former disease, as there is unquestionably in the latter. Is not the mere fact of the curvature *redressing* itself when the person is in the horizontal position, and therefore when all the weight of the head and neck is withdrawn from the weak spinal column, a sufficient evidence of this fact? We think it is.

Club-foot is never the result, as far as we know, of weakness in the affected part. But it is unnecessary to say more on this subject at present, as the pathological reasonings of M. *Guerin* are much less likely to be adopted than his manipulations. His practice of dividing some of the spinal muscles has been tried by one or two surgeons in this country; and, it may be, with a certain amount of success in a few cases. But we must protest against the operation being at all applicable, in a general point of view, to the treatment of lateral curvature of the spine; for the practice is based on erroneous principles, and cannot therefore be successful in the majority of instances.

What has been said above will serve, among other examples, to guard the medical reader from hastily yielding his belief to all the bold assertions of popularity-hunting doctors, whether in our own country or on the continent. There is no end to clap trap novelties in our profession. On the one hand, we have men who cure all deformities, whether of the eyes, or the limbs, or the back, by cutting muscles and tendons, and would make us believe that stammering and deafness may be got rid of almost *instanter* by snipping off a bit of the uvula or tonsils—(by the bye, what is Mr. *Yearsley* doing now-a-days?)—and on the other, we have at the present moment quite a bevy of *claqueurs*—including metropolitan surgeons, and Irish-dubbed knights—discoursing most learnedly on the merits of Hydropathy, and hospital physicians and country parsons proclaiming the wonders of Animal Magnetism.

Truly this is the age of rampant quackery; and high time it is that those, who value the dignity of professional credit, should unhesitatingly denounce the folly or the knavery—for assuredly it is one or the other—of all such practices.—*Lond. Medico-Chirurgical Review, Jan.* 1844.

